# Opportunities toward Scalable Domain-Wall-Based Spintronic Devices

**DOI:** 10.1021/acsnanoscienceau.6c00113

**Published:** 2026-09-09

**Authors:** Jae-Chun Jeon, Lukas Fischer, Yung-Cheng Li, Renata Lei, Stuart S. P. Parkin

**Affiliations:** Max Planck Institute of Microstructure Physics, Weinberg 2, 06120 Halle (Saale), Germany

**Keywords:** racetrack memory, domain-wall dynamics, 3D spintronics, computing architecture, nanoscale spintronics

## Abstract

Since its initial proposal in 2004, racetrack memory, which utilizes current-induced motion of domain walls within a magnetic nanowire, has evolved from a conceptual spintronic device into a mature research platform supported by major advances in materials engineering, nanotechnology, domain-wall physics, and spin-torque mechanisms. Yet, practical racetrack memory or logic remains challenging. The field has entered a new state in which the dominant challenges are no longer associated with driving domain walls or understanding the fundamental mechanisms behind them, but with achieving reliable device operation and fabrication of practical nanoscale devices. Key bottlenecks include the electrical readout of nanoscopic racetrack devices, the integration of magnetic tunnel junctions, the control of stochastic domain-wall behavior, positioning accuracy, and scalability toward three-dimensional (3D) architectures. We briefly summarize the current status of the field and discuss how these challenges redefine research priorities while creating opportunities for advances in electrical readout, 3D device integration, and novel domain-wall-based computing architectures. By highlighting emerging directions in device engineering and advanced characterization, we outline pathways toward translating laboratory-scale demonstrations into scalable, manufacturable spintronic memory and logic technologies.

## Graphical abstract

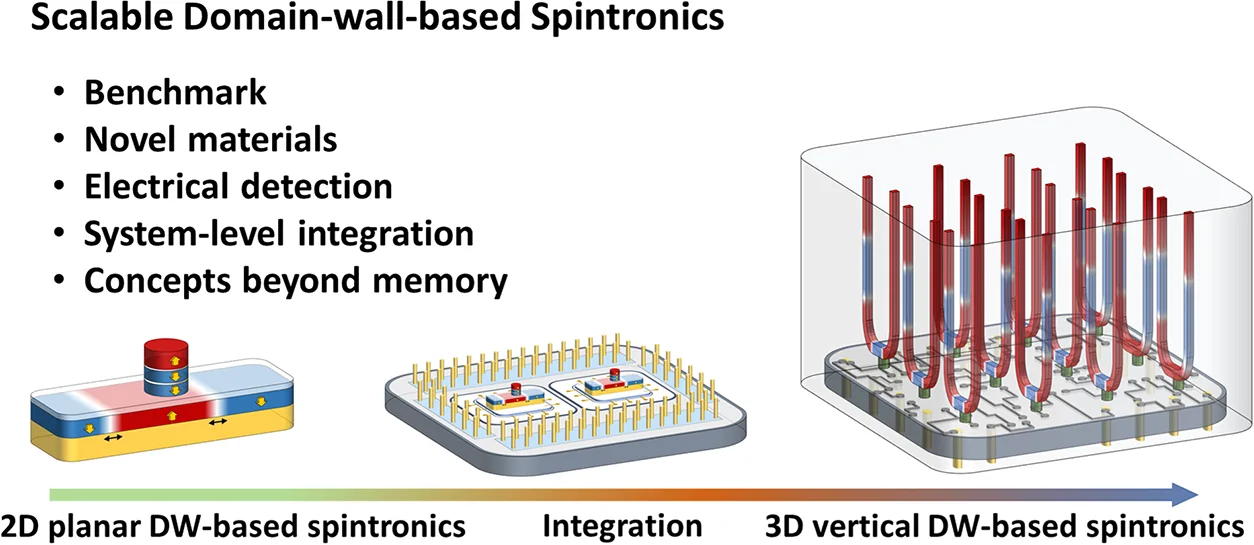

## 1 Introduction

As complementary metal–oxide–semiconductor (CMOS) device dimensions approach fundamental physical limits, alternative information-processing technologies have attracted increasing attention with the rapidly increasing demand for memory, driven by the accelerating development of artificial intelligence.^1^ Among the candidates, spintronics offers a particularly attractive route because it exploits the electron spin degree of freedom in addition to charge, enabling nonvolatility, high-speed operation, and potentially lower energy consumption.^2–4^

Within the broad landscape of spintronic technologies, domain-wall-based spintronic memory, i.e., racetrack memory, occupies a unique position. Originally, the concept was proposed by Parkin as a shift-register-based memory architecture in which magnetic domain-walls store information and are transported along nanowires by electrical currents.^5^ Racetrack memory promised an unusual combination of characteristics: nonvolatility, high endurance, fast operation, and three-dimensional (3D) scalability.^5,6^ Unlike conventional magnetic memories, where only one bit of information is stored per memory cell, racetrack memory relies on the current-induced motion of domain walls along a single magnetic track – allowing multi-bit storage in a single racetrack memory cell. The mechanism will be discussed in detail later. Figure 1a illustrates a key driving mechanism of current-induced domain-wall motion in a heavy-metal (HM)-ferromagnet (FM) bilayer system, which is a key base structure for racetrack devices. In such a system, when a charge current flows through the heavy metal layer, a spin current is generated via the spin Hall effect and transfers angular momentum to the adjacent ferromagnet. The interaction between this spin current and the domain-wall, particularly a chiral Néel wall, exerts a spin torque that induces domain-wall motion. The racetrack architecture offers the possibility of significantly increasing storage density while reducing the footprint of peripheral circuitry. Figure 1b illustrates the original proposal of racetrack memories based on a ferromagnetic nanowire integrated with a read sensor (magnetic tunnel junction, MTJ) and a write port (Oersted field) in a 3D structure.^6^ A similar structure was proposed using synthetic antiferromagnetic nanowires.^7^

**Figure 1:**
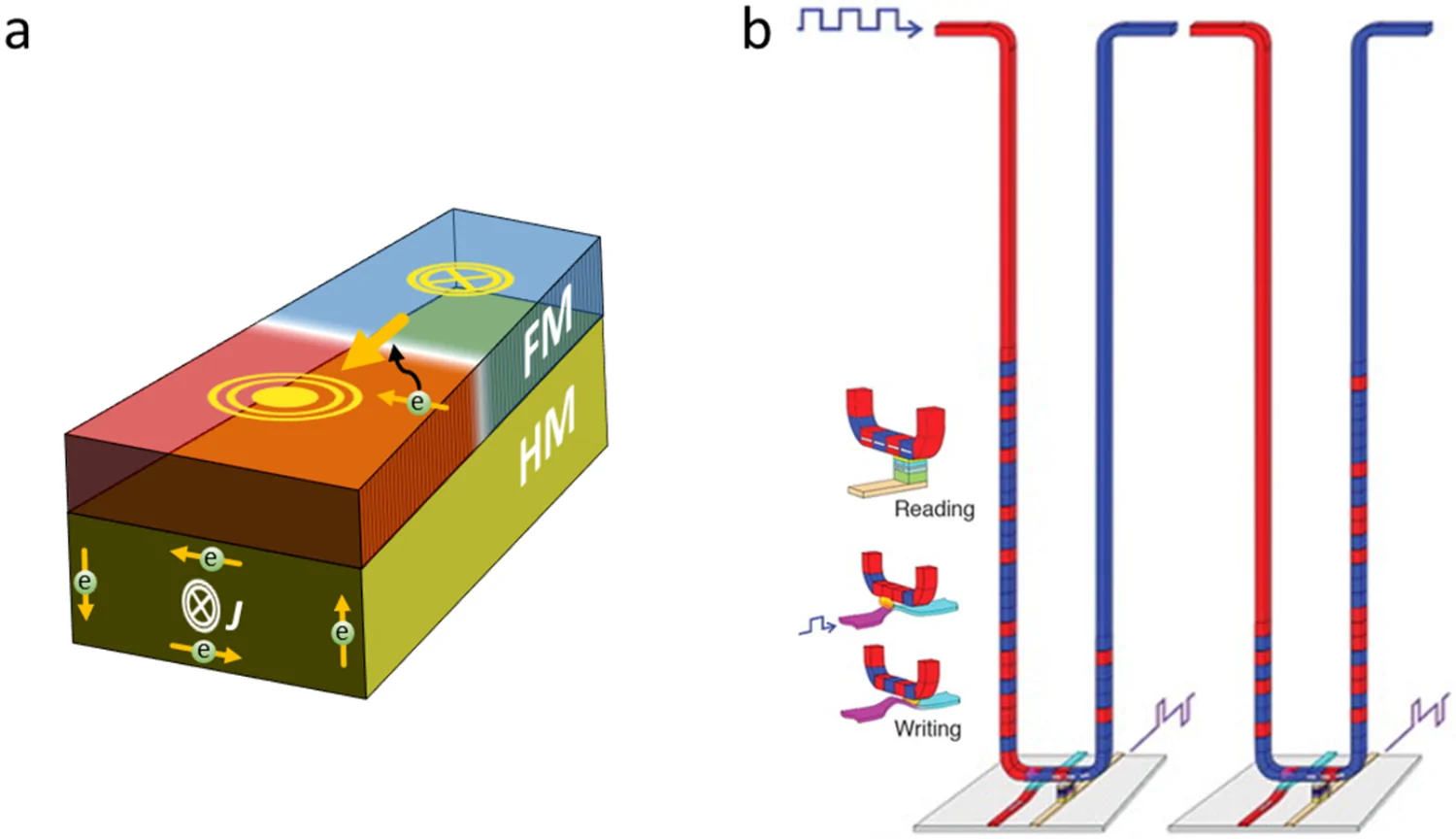
Concept of racetrack memory. (a) Illustration of current-induced domain-wall motion in a heavy metal (HM)-ferromagnet (FM) bilayer system with a Néel domain wall. Spin current generated in the HM is injected into the adjacent FM layer and interacts with the domain wall, generating spin torque that drives the motion of the domain wall. (b) Original concept of a ferromagnet-based three-dimensional racetrack memory – it consists of bit-storage element (ferromagnetic nanowire), a read sensor (magnetic tunnel junction, MTJ), and a write port (Oersted-field). Reproduced with permission from ref 6 Copyright [2008] Science.

The original concept generated enormous interest and stimulated a wide range of related research because it linked fundamental spintronic phenomena with an ambitious technological vision. At the time of its proposal, several key scientific questions remained unresolved. Reliable manipulation of domain walls at practical current densities was complicated by defect-induced pinning, thermal fluctuations, and stochastic variations in domain-wall motion, which limited deterministic control. Meanwhile, efficient methods for reading and writing information within racetrack structures were still under development. The first decade of racetrack-related research was dominated by efforts to control domain-wall motion and understand the mechanisms behind. These investigations have led to the discovery of novel and intriguing physical phenomena in spintronic devices. Researchers identified the limitations of field-driven motion, established spin-transfer torque (STT) as an effective driving mechanism, and later demonstrated the advantages of spin-orbit torques (SOTs) generated in HM-FM heterostructures, which are now considered as a fundamental building block for spintronics.^8–13^ Simultaneously, advances in materials engineering enabled the transition from in-plane magnetic systems supporting vortex domain-walls to perpendicularly magnetized multilayers hosting chiral Néel walls.^14–16^ The discovery and exploitation of interfacial Dzyaloshinskii–Moriya interactions (DMIs) in the relevant material systems further transformed the field by stabilizing domain-wall structures with improved dynamic properties.^12,13^

As a result, the achievable domain-wall velocities increased dramatically, energy consumption decreased, and the fundamental understanding of current-driven magnetic textures matured. Synthetic antiferromagnets, chiral kagome antiferromagnets, and ferrimagnets subsequently emerged as attractive platforms capable of suppressing stray fields, reducing current density requirements, and mitigating dynamic instabilities.^17–19^ In particular, the reduction in current density is relatively well established in ferrimagnets with the angular momentum compensation,^20–27^ whereas similar improvements in synthetic antiferromagnets^17,28–35^ and chiral kagome antiferromagnets^19,36^ have been demonstrated under specific materials rather than being universally applicable. From a fundamental scientific perspective, many of the obstacles that initially motivated racetrack research have thus been addressed significantly. However, technological success requires more than demonstrating efficient domain-wall motion in laboratory-level micron-size devices that are accessible to conventional magneto-optical imaging techniques.^12,13,38,39^ Many of the technologically relevant phenomena occur at the nanometer scale. Our understanding of domain-wall behavior at such scales remains surprisingly limited despite two decades of intensive research. A memory technology must satisfy a complex set of requirements, including reliable operation over millions or billions of cycles, precise control of domain-wall position, thermal budget, field immunity, compatibility with large-scale manufacturing, and efficient integration with peripheral circuitry.^3,40,41^ When viewed from this point, the critical challenges facing racetrack memory today differ substantially from those that shaped its early development.

In this perspective, we will briefly summarize key advances of materials and devices in racetrack memory and discuss the opportunities towards realizing practical nanoscopic domain-wall-based spintronic devices. The emphasis of future research must increasingly shift toward nanoscale reliability, direct electrical readout, device integration, 3D device fabrication, and system-level performance. While fundamental spintronic physics will continue to play an essential role to explore novel materials and innovative device concepts, the next step is likely to depend more strongly on overcoming practical implementation challenges than on discovering new mechanisms for accelerating domain-wall motion.

## 2 Progress in Racetrack Technologies: Evolution of Magnetic Materials and Driving Mechanisms

The development of magnetic materials and atomically engineered thin films has been central to domain-wall-based spintronic devices. Early studies primarily used soft magnetic materials such as permalloy (Py), where domain walls could be generated and manipulated with relative ease. Initial research focused on magnetic-field-driven domain-wall motion because of its experimental simplicity. However, field-based approaches are poorly suited for practical applications due to scalability challenges and high energy consumption. The introduction of STT enabled direct electrical control of domain walls through spin-polarized currents, eliminating the need for external magnetic fields.^6,8–11,14,42^ Although high current densities were still required (threshold current density, *J*_th_ > 50∼100 MA cm^–2^ with *v* ∼ 100 m s^–1^),^8,10,11,14^ STT established the foundation for electrically driven racetrack devices. Figure 2a demonstrates current-induced domain-wall motion in a Py nanowire.^6^

**Figure 2:**
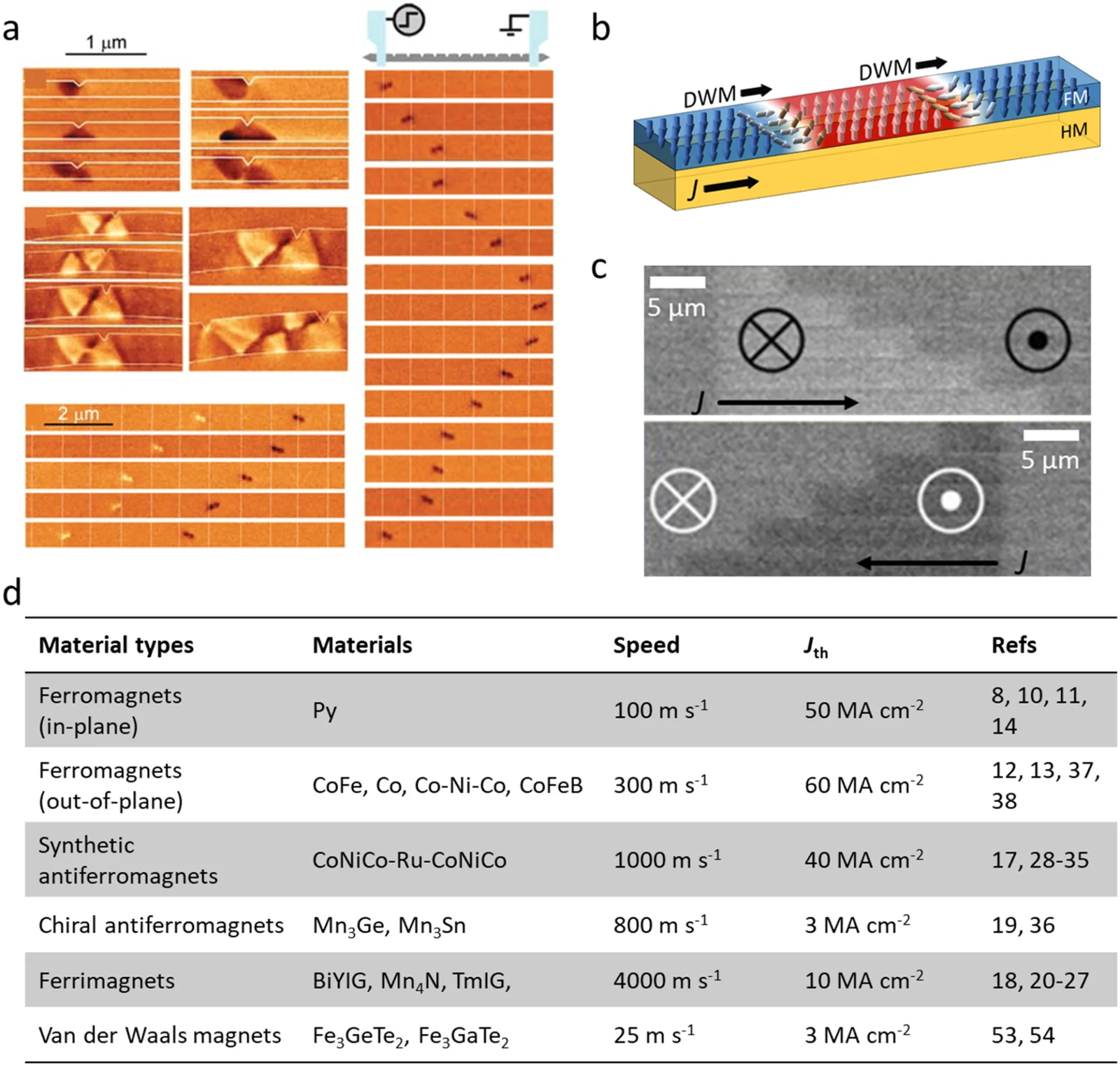
Evolution of materials and understanding of driving mechanisms. (a) Magnetic force microscopy (MFM) images showing current-induced domain-wall motion in Permalloy nanowires. Reproduced with permission from ref 6 Copyright [2008] Science. (b) Schematic illustration of the driving mechanism of current-induced domain-wall motion in a FM-HM heterostructure, where the DMI-induced Néel domain wall plays a key role in efficient motion. (c) Kerr microscope images of current-induced domain-wall motion in an atomically engineered synthetic antiferromagnetic racetrack, where maximum domain-wall velocity reaches 1000 m s^–1^. Reproduced with permission from ref 47 Copyright [2001] John Wiley and Sons. (d) Representative magnetic materials and their corresponding domain-wall velocities and threshold current densities (*J*_th_) for current-induced domain-wall motion.

The introduction of ferromagnets with perpendicular magnetic anisotropy (PMA) and spin Hall effect (SHE) represented another major turning point.^12,13,16,43^ PMA ferromagnetic materials host narrower domain-walls, establish improved thermal stability, and enable higher storage densities. More importantly, PMA systems provided a favorable environment for exploiting interfacial spin-orbit effects that later became central to current-induced domain-wall motion. The emergence of SOT further transformed the field. HM-FM heterostructures such as Pt-Co, Ta-CoFeB, and W-CoFeB established a versatile materials platform in which interfacial spin-orbit coupling generated efficient current-induced torques.^12,13,37,38^ Concurrently, as illustrated in Figure 2b, interfacial DMI stabilized well-defined chiral Néel walls that allows for deterministic and highly efficient current-induced domain-wall motion.^12,13,43,44^ SOT-based racetrack devices with PMA typically exhibit a *J*_th_ ∼ 50 MA cm^–2^ with a maximum domain-wall velocity exceeding 300 m s^–1^ (at *J* > 200 MA cm^–2^).^12,13,37,38^

Later, synthetic antiferromagnets (SAFs) have garnered considerable attention due to their desirable characteristics for racetrack applications. By antiferromagnetically coupling two ferromagnetic layers through a nonmagnetic spacer (typically Ru) via long-range oscillatory interlayer exchange coupling, these systems combine many of the advantages of natural antiferromagnets with the practical accessibility of ferromagnetic materials.^17,45,46^ In particular, using nanosecond current pulses, domain-wall velocities reach up to 750 m s^–1^, substantially higher than in comparable ferromagnetic systems while maintaining a similar *J*_th_ of ∼40 MA cm^–2^.^17^ Figure 2c shows differential Kerr images of current-induced domain-wall motion in an atomically engineered SAF racetrack, where domain-wall velocities approaching near 1000 m s^–1^ are achieved.^47^ Numerous following works have defined that SAF racetracks as a promising platform for high-density, low-stray-field, and ultrafast spintronic devices, overcoming major limitations of conventional racetrack memories and enabling more efficient domain-wall-based information storage.^28–35,47^

Another important class of magnetic materials for racetrack memory is ferrimagnets, in which two or more magnetic sublattices are coupled antiferromagnetically but have unequal magnetic moments, resulting in a nonzero net magnetization.^20^ Ferrimagnets may exhibit much faster domain-wall motion due to their reduced total angular momentum and magnetic stray fields, as well as their increased exchange field.^18,21–27^ Near angular momentum compensation conditions, ferrimagnetic systems can exhibit exceptionally fast domain-wall motion (>4000 m s^–1^, *J*_th_ ∼ 10 MA cm^–2^) while maintaining efficient electrical controllability.^18^ These properties suggest promising opportunities for high-speed racetrack devices.

Recently, researchers have demonstrated that noncollinear chiral antiferromagnets (NCAFs), i.e., Mn_3_Ge and Mn_3_Sn, exhibit high-velocity current-induced domain-wall motion. Unlike conventional materials, NCAFs possess very complex domain-wall structure originated from the cluster magnetic octupole order associated with the noncollinear spin texture and broken time-reversal symmetry.^19,36,48,49^ In bulk Mn_3_Sn, the domain-walls, aligned parallel to the kagome planes, exhibit Bloch-like spin textures in which the magnetic octupole moment rotates continuously between domains. Current densities of only ∼10^9^ A m^–2^ were sufficient to nucleate and move the walls, significantly lower than those typically required in ferromagnets (typically ∼10^11^ A m^–2^).^36^ More recently, by engineering Bloch- and Néel-type walls in single crystal Mn_3_Sn and Mn_3_Ge, efficient motion is observed, with Néel walls reaching velocities up to 750 m s^–1^ at current densities below 10^11^ A m^–2^.^19^ These studies, together with recent demonstration of MTJ integration using Mn_3_Pt and Mn_3_Sn with its electrical control in Mn_3_Pt,^50–52^ establish noncollinear antiferromagnets as promising platforms for fast, energy-efficient, and electrically controlled domain-wall devices.

Figure 2d lists and summarizes representative material types, materials (multilayer stacks and van der Waals materials),^53,54^ their corresponding near-maximum domain-wall velocities, and threshold current densities. The rapid success of these approaches with various materials has shifted the bottleneck away from domain-wall motion efficiency itself. Domain-wall velocities exceeding hundreds of meters per second are routinely reported, and in some material systems the values are even reaching a few thousand meters per second. Due to this successful enhancement of the attainable domain wall velocity and positioning efficiency to technologically relevant scales, further progress of domain-wall based spintronic device is now increasingly limited by other factors than the domain-wall velocity.

## 3 From Fundamental Physics to Technology

Racetrack memory and related spintronic technologies have been dramatically improved over the last two decades. Initially, the research was dominated by fundamental questions regarding the nature of domain-wall motion in ferromagnetic materials. During this period, researchers sought to understand how magnetic textures respond to external fields, spin-polarized currents, and SOTs. Many of the key discoveries that now constitute the foundation of modern spintronics, including current-induced domain-wall motion, chiral magnetic textures, interfacial DMIs, and spin Hall effects, were either motivated by or closely connected to racetrack-related research.

Today, however, the field appears to be entering a new phase. Rather than exploring domain-wall motion itself, researchers are raising questions concerning whether domain-walls can be controlled reliably within realistic memory or logic devices. Demonstrating a physical phenomenon under carefully controlled laboratory conditions in relatively large systems, i.e., micron-sized devices, is fundamentally different from implementing a technology capable of competing with established memory platforms on a deep sub-micron scale in a system-level. A useful comparison can be made with STT-based magnetic random-access memory (STT-MRAM). The underlying physics of STT was established years before commercially viable devices emerged.^55,56^ The eventual success of MRAM was not driven solely by improvements in switching mechanisms but by correlated advances in process integration, device reliability, materials uniformity, and manufacturing compatibility.^57^ Similar challenges are likely to shape the future development of domain-wall-based memory (DWM) as a viable technology. Alongside DWM, several important emerging nonvolatile memory technologies have attracted significant attention, including resistive-switching random-access memory (RRAM),^58–61^ phase-change random-access memory (PCRAM),^62–65^ ferroelectric random-access memory (FeRAM),^66–69^ and SOT magnetic random-access memory (SOT-MRAM).^57,70–73^ While each of these technologies offers distinct advantages, DWM presents unique opportunities in terms of memory density and operational performance. Table 1 compares the key characteristics of these emerging memory technologies, including cell density, number of terminals, endurance, latency, energy consumption, CMOS compatibility, thermal budget, and technology maturity, providing a concise overview of the potential advantages of DWM. At its core, DWM shares the basic film stack and device structure of SOT- or STT-MTJ-based memories. In this sense, DWM can be viewed as an extension of SOT/STT-MTJ concepts, in which domain walls are nucleated and manipulated within an elongated free layer. This geometry enables information to be stored along the free layer and can effectively reduce the energy required for memory operation while enabling field-free switching.

**Table 1:** Comparison of Emerging Nonvolatile Memory Technologies

parameter	STT-MRAM	SOT-MRAM	RRAM	PCRAM	FeRAM	DWM
cell density	20–40 F^2^	60–100 F^2^	4–8 F^2^	4–8 F^2^	>20 F^2^	<4 F^2^
terminals	2	3	2	2	2	2 or 3 (by design)
endurance	10^12^–10^15^ cycles	>10^15^ cycles	10^6^–10^12^ cycles	10^7^–10^9^ cycles	10^14^–10^16^ cycles	not explored
read latency	5–20 ns	2–10 ns	10–100 ns	20–100 ns	20–100 ns	= SOT-MRAM
write latency	5–20 ns	1–5 ns	10–100 ns	50–500 ns	20–100 ns	1–5 ns
write energy	10–100 fJ/bit	1–20 fJ/bit	1–100 fJ/bit	∼100 fJ/bit	1–10 fJ/bit	< SOT-MRAM
CMOS compatibility	excellent (BEOL compatible)	excellent (= STT-MRAM)	good	good	moderate	excellent (= SOT-MRAM)
thermal budget	moderate 300–400 °C	= STT-MRAM	low-moderate <400 °C	high >400 °C	low-moderate <400 °C	= SOT-MRAM
maturity	commercial (high)	prototype	prototype/near commercial	commercial (limited)	commercial (limited)	research stage

Many performance metrics commonly reported in racetrack studies, including maximum domain-wall velocity, threshold current density, domain wall mobility, and depinning efficiency, provide valuable insights into underlying physics but only partially reflect technological relevance. While these metrics primarily characterize the intrinsic domain-wall dynamics, the practical implementation of current-driven domain-wall devices requires careful consideration of several nonideal effects, including Joule heating, local defects, repeatability, device-to-device variability, and domain-wall stability. These factors become increasingly significant at high current densities and can substantially influence the measured domain-wall dynamics and switching behavior, potentially leading to an overestimation of device performance if they are not properly accounted for. For practical applications, metrics such as bit-error rate (BER), wall-to-wall spacing, positional accuracy, retention, endurance, and device-to-device variability in nanoscopic racetrack devices may ultimately be more important.^74–79^ However, these issues have received surprisingly little attention.

This shift in emphasis requires a corresponding change in experimental methodology. So far, the field has heavily relied on optical imaging techniques that provide direct visualization of magnetic structures in a micron dimension. While these approaches have been indispensable for understanding fundamental physics, they often operate on dimensions substantially larger (typically 2∼10 μm) than those desired for commercial devices (sub-100 nm). Therefore, research should apply methodologies capable of evaluating statistically meaningful device populations at technologically relevant scales. Furthermore, the transition from individual device demonstrations to integrated circuits on a system-level introduces additional constraints that are rarely considered in fundamental studies. Current distribution, thermal management, process variation, interconnect overhead, and compatibility with back-end-of-line fabrication processes all become critical factors. As a result, some material systems that appear highly attractive from a physics perspective may ultimately prove difficult to implement in the context of large-scale manufacturing environments.

The success of racetrack memory will depend on a broader and more interdisciplinary approach that combines spintronics, materials science, device engineering, circuit design, and manufacturing science. The central challenge is no longer simply discovering new physical effects but integrating existing knowledge into a coherent, robust technological platform.

## 4 Technological Challenges and Perspectives

### 4.1 Direct Electrical Read from Nanoscopic Racetrack Devices

Among the numerous challenges facing racetrack memory, electrical readout remains one of the most important and least appreciated. In many laboratory demonstrations, domain-wall motion is characterized using either imaging techniques such as magneto-optical Kerr microscopy, magnetic force microscopy, and X-ray microscopy or low-fidelity electrical transport measurement techniques such as magnetoresistance and Hall effect.^6,8,10,12,13,80^ These methods provide exceptional insight into magnetic behavior but are not practical for information technologies.

From a technological perspective, a functional memory device requires an integrated electrical mechanism capable of reading the magnetic state with high fidelity, low energy consumption, low noise, and high throughput. MTJs naturally emerge as the most attractive solution because they have already demonstrated technological viability in commercial MRAM products. Integrating MTJs directly with racetrack structures could potentially leverage decades of progress in magnetic sensing and spintronic device engineering. From the conceptual perspective, MTJ integration in racetrack appears straightforward. An MTJ positioned above a racetrack can detect the local magnetization through tunnel magnetoresistance, enabling electrical readout of passing domains. In practice, however, the situation is considerably more complicated.

Only a limited number of research groups have demonstrated the integration of magnetic tunnel junctions (MTJs) with racetrack devices. Figure 3a–3c presents representative examples of racetrack devices, in which current-induced domain wall motion is electrically detected using integrated MTJs.^81–83^ For brevity, not all studies can be discussed here; nevertheless, several additional works have also reported optimistic results for MTJ-integrated racetrack architectures.^72,73,76,84^ Unfortunately, the number of available studies remains insufficient to establish statistically meaningful trends. One major reason is the high degree of complexity associated with fabricating nanoscopic devices. In the following sections, we discuss major challenges that must be addressed.

**Figure 3:**
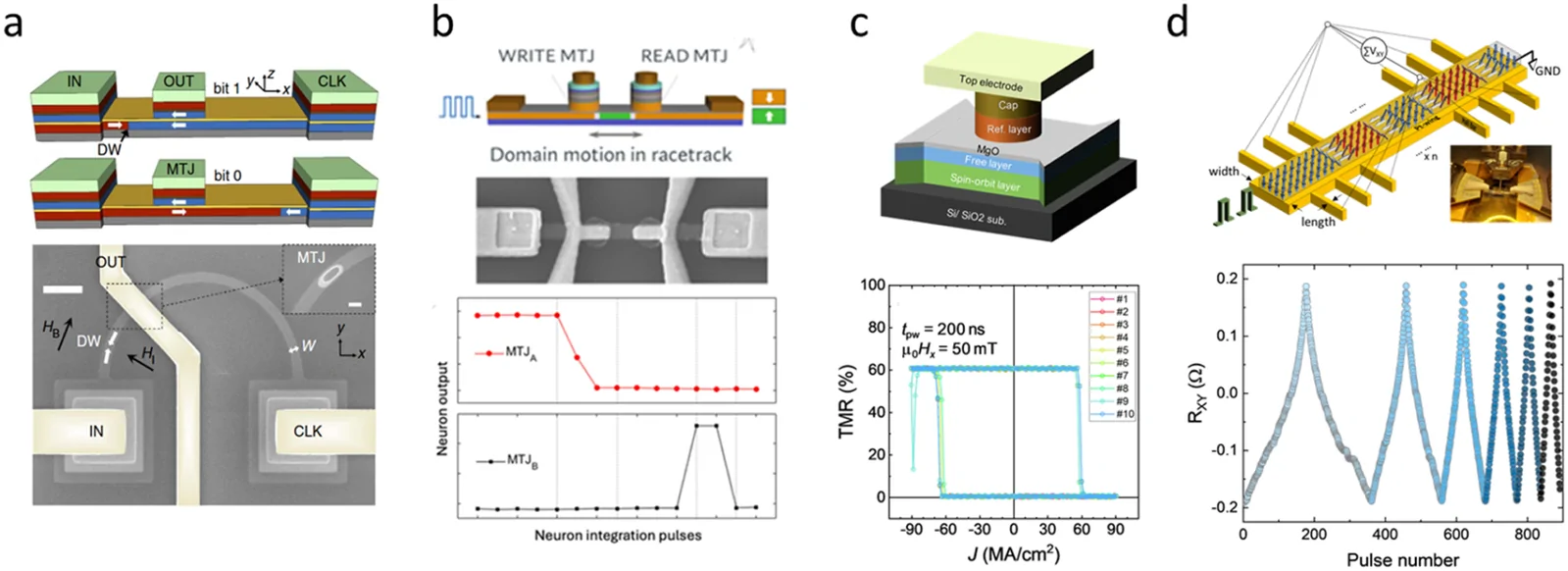
Demonstrations of advanced electrical detection techniques. (a) Demonstration of a prototype logic device based on three-terminal MTJs with mobile domain walls in an in-plane CoFeB-based racetrack. Reproduced from^81^. Available under a CC-BY 4.0 license. Copyright [2016] Springer Nature. (b) Demonstration of integrate-and-fire behavior using domain-wall motion in nanoscopic MTJs integrated into a racetrack device with perpendicular magnetic anisotropy. Reproduced from^82^ Copyright [2025] American Chemical Society. (c) Electrical writing of domain walls in an MTJ-integrated racetrack device. In this work, a hybrid Pt–Co/CoFeB structure is used as the free layer, which enables both efficient current-induced domain wall motion and a reasonably high TMR. Reproduced from^83^. Available under a CC-BY-NC-ND 4.0 license. Copyright [2025] Springer Nature. (d) Multicore memristor architecture based on electrically readable nanoscopic racetrack devices. In such a device design, domain wall dynamics in sub-100 nm devices were investigated with proposed analyses schemes. Reproduced with permission from ref 103 Copyright [2024] Science.

#### 4.1.1 Compatible Multilayer Stacks for Both Current-Induced Domain-Wall Motion and Tunneling Magnetoresistance

Identifying material systems that simultaneously support efficient current-induced domain-wall motion and high tunneling magnetoresistance (TMR) is a key aspect. In conventional CoFeB-based MTJ film stacks, post-annealing process is essential for achieving a high TMR, as it promotes crystallization of the initially amorphous CoFeB layers and enables coherent tunneling through the crystalline MgO barrier.^85–87^ However, many materials commonly employed in racetrack devices, such as Co, CoGd, TmIG, and Py, are susceptible to degradation during the post-annealing process and do not provide the favorable crystallographic matching with the MgO tunnel barrier required for efficient coherent tunneling. These limitations pose challenges to integrating these materials with optimized CoFeB/MgO-based MTJs exhibiting high TMR values (>100%).

Therefore, it may seem natural to develop racetrack memory devices based on heavy-metal/CoFeB bilayer structures, given the maturity of these film stacks and their established compatibility with high TMR.^88–90^ However, current-induced domain-wall motion in CoFeB can be highly sensitive to extrinsic pinning and microstructural variations, including edge roughness, interfacial disorder, and thickness inhomogeneity, which can lead to variations in the depinning current and propagation dynamics.^91–93^ In addition, their relatively weak DMI and magnetic anisotropy make them more susceptible to stray-field and thermal effects arising from uncompensated fields from the reference pinning layer and current-induced Joule heating, which can further modify the domain-wall configuration and pinning landscape.^93–95^ As such, although optimized CoFeB-based heterostructures can exhibit efficient and weakly pinned domain-wall motion, achieving highly stable and reproducible dynamics requires careful control of the interfaces, microstructure, device geometry, and thermal conditions. To circumvent these challenges associated with the delicate optimization of CoFeB-based racetracks, Raymenants et al. (IMEC)^96^ and Okada et al. (Samsung Electronics)^83^ have demonstrated hybrid synthetic antiferromagnetic structures in which Co or Co–Ni–Co and CoFeB layers are exchange-coupled using a Ru spacer, enabling both efficient current-induced domain-wall motion and reasonable TMR on/off ratios (>60%). Nevertheless, fabrication compatibility remains a significant challenge for such multilayer stacks. State-of-the-art MTJs with high TMR (>150%) typically require highly optimized multilayer stacks and annealing processes. While the overall multilayer stacks may be similar to those used in conventional STT- or SOT-MTJs, simultaneously achieving efficient current-induced domain-wall motion and high TMR remains nontrivial. Thermal budgets, interface quality, layer roughness, and patterning-induced damage must all be carefully controlled.

#### 4.1.2 Process Window of Device Fabrication

The precision of the fabrication process which integrates direct read sensor is another critical aspect. As demonstrated in recent studies, damage to the free layer (i.e., the racetrack) appears difficult to avoid during device fabrication process.^83,96^ Because the etching process must stop precisely at the MgO layer, energetic ion beams can damage the underlying free layer. Samsung Electronics reported that post-fabrication annealing process (300 °C for 1 Hr under vacuum) could partially recover such damage.^83^ For reliable, high-yield manufacturability at large wafer scales (e.g., 300 mm), a uniform and damage-free etching process is essential. Multi-angle ion-beam etching with multi-level control of the ion-beam energy should be developed in order to maximize the process window. In addition to accurate etch-depth control, several challenges associated with large-scale nanoscopic device fabrication must be carefully addressed, particularly in the case of MTJs. These include re-deposition of etched materials, side-wall cleaning, resist hardening during ion-beam etching, and the complete removal of residual resist. An alternative approach is to avoid such etching process for direct reading and sense the stray field generated by the racetrack. Recently, Quinsat et al. demonstrated a reliable magnetic shift register based on remote stray-field sensing.^74^ However, it should be noted that such remote sensing is only applicable to magnetic layers that generate sufficiently strong stray fields. In the case of SAFs, which are highly desirable for achieving high-density, high-speed, and enhanced stability, remote sensing does not seem directly applicable because of their minimal stray fields. Another strategy to mitigate ion-beam damage is to fabricate an MTJ that covers the entire racetrack. Such a configuration is expected to produce analog-like output signals, which may be advantageous for unconventional computing paradigms, including neuromorphic computing.^97^ Table 2 summarizes different types of MTJ integrated racetrack devices, along with their expected outputs and associated challenges. Furthermore, the requirement to the fabrication alignment between racetrack MTJ become increasingly challenging as device dimensions shrink. The relative position between the sensing component (MTJ) and the moving domain-walls within the racetrack directly influences signal amplitude and readout reliability. For example, if one designs 50 nm MTJ on 70 nm wide racetrack, there is only about 10 nm of acceptable margin for the device fabrication. In such dimensions, nanometer-scale misalignment may lead to substantial device-to-device variations and degraded performance.

**Table 2:**
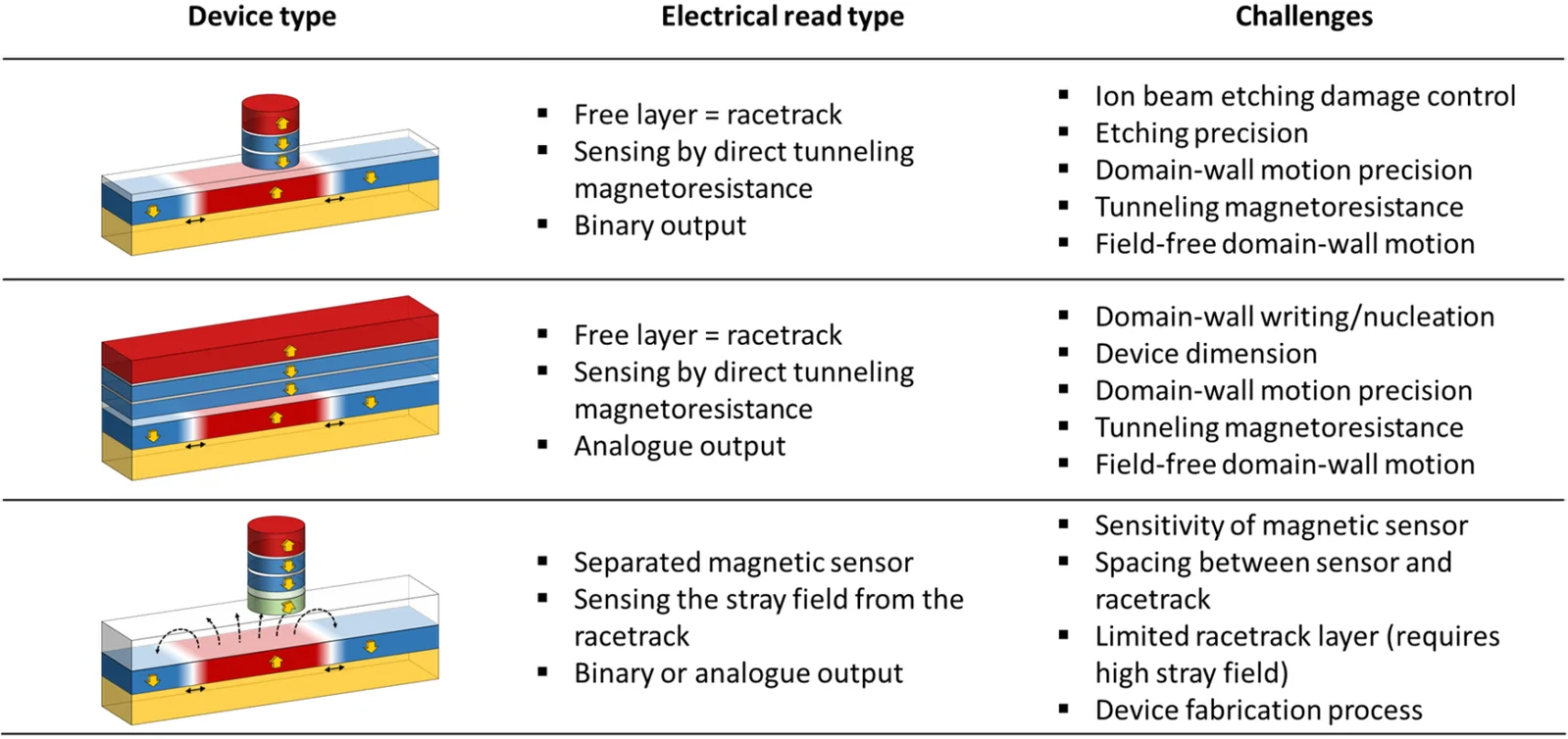
Challenges and Opportunities for Different Types of Racetrack Memory Devices

#### 4.1.3 Field Immunity

Field immunity represents another important challenge and is a common concern in magnetic sensor technologies. External magnetic fields, as well as stray fields originating from adjacent magnetic components, can modify the local energy landscape and consequently influence domain-wall (DW) dynamics. Experimentally, DW propagation in magnetic racetracks has been shown to exhibit strong field-dependent stochasticity, reflecting the competition between the driving field and the spatially varying pinning landscape.^98,99^ In addition, the stray field generated by a magnetic reference layer in an MTJ can modify DW-mediated switching dynamics and, particularly at reduced device dimensions, induce oscillatory or delayed switching behavior.^100,101^ These effects can become increasingly important as the characteristic device dimensions approach the DW width, where local magnetic interactions are no longer negligible. For example, stray fields generated by the MTJ reference layer may alter the local energy landscape, creating unintended pinning sites, perturbing domain-wall trajectories, or even destabilizing neighboring domain-walls.

One promising strategy to mitigate such parasitic magnetic interactions is the use of synthetic antiferromagnets (SAFs) or natural antiferromagnets (AF). The negligible magnetic moments of SAF or AF structures substantially reduce stray fields while maintaining strong interlayer exchange coupling, making them attractive for densely packed racetracks. Experimental and theoretical studies have shown that SAF racetracks can suppress DW tilting and enable closely spaced DWs, providing advantages for high-density and robust DW transport. Therefore, ultimately, all-antiferromagnetic architectures, in which both the information-carrying layer and the sensing element are based on SAFs or natural antiferromagnets,^17,19,36,76,102^ could provide an attractive route toward field-immune DW devices.

#### 4.1.4 Domain-Wall Dynamics at the Nanoscale

An additional complication arises from the dynamic nature of racetrack operation. Unlike conventional MRAM, where magnetic states remain stationary during readout, racetrack memory devices store information in magnetic domains and perform read and write operations through the current-induced motion of domain walls. Consequently, sensing mechanisms must operate reliably under dynamic conditions while providing sufficient temporal and spatial resolution to distinguish neighboring domains. This requirement becomes increasingly challenging as the DW spacing is reduced, since closely packed DWs can become difficult to resolve electrically. A recent experiment has demonstrated strategies to address this challenge by dynamically manipulating DW spacing, enabling the compression and decompression of neighboring DWs to facilitate electrical readout while retaining high data density.^76^ Importantly, advances in integrated electrical sensing have demonstrated that dynamic DW motion can be tracked directly at the nanoscale. Jeon et al. experimentally demonstrated electrical tracking of multiple mobile DWs in nanoscopic racetracks using integrated anomalous Hall detectors, achieving a spatial resolution better than 40 nm.^103^ The time-dependent electrical signals further enabled the analysis of both the static and dynamic behavior of individual and multiple DWs, providing a route to quantify their stochastic dynamics during operation. These results provide strong evidence that integrated electrical detectors can resolve DW dynamics in deeply downscaled racetracks, while also highlighting the importance of temporal signal analysis for evaluating dynamic reliability. Beyond Hall-based detection,^103–107^ electrically readable racetracks have recently demonstrated precise positioning of multiple DWs at sub-micrometer pinning sites and analog-like output signals, further illustrating the possibility of extracting more than binary information from the dynamic magnetic state.^103,107–110^ Such capabilities could be particularly important for future nonbinary,^103,108^ neuromorphic,^97,108–110^ and probabilistic racetrack architectures,^111^ where the temporal evolution of the electrical signal itself may constitute part of the computational information. Thus, future sensing schemes should not only maximize spatial resolution but also capture the temporal statistics, stochasticity, and correlations associated with DW motion.

### 4.2 Reliability and Stochasticity (Noise and Thermal Fluctuations)

Reliable operation requires predictable domain-wall motion over millions or billions of cycles. However, domain-walls are inherently sensitive to imperfections in the magnetic landscape. Variations in anisotropy, interfacial roughness, grain structure, and local defects can all influence dynamic behavior. At micron scales, these effects often appear as minor perturbations. At nanometer dimensions, however, small variations in local material properties can produce substantial fluctuations in depinning currents, propagation velocities, and stopping positions. In fact, real-time measurements have demonstrated broad distributions of both DW velocity and propagation distance under nominally identical driving conditions, with local pinning and dynamic effects producing pronounced stochasticity.^99^ Direct measurements of DW depinning have further shown that the depinning field itself exhibits a statistical distribution that depends on nanowire geometry, including wire width and notch dimensions.^112^ As a result, stochasticity may become a defining characteristic of nanoscale racetrack devices. Understanding the statistical nature of domain-wall motion in nanoscopic devices is therefore likely to be as important as understanding its average behavior. Studies should therefore systematically quantify failure probabilities, error distributions, and cycle-to-cycle variability. Such information is essential for evaluating technological feasibility but remains surprisingly scarce in the literature.

Furthermore, thermal fluctuations are unavoidable in any nanoscale system. Their influence becomes particularly significant when the thermal energy approaches characteristic magnetic energy scale. In racetrack memory devices, thermal effects can affect depinning probabilities, modify propagation dynamics, and contribute to positioning uncertainty.^110^ Experimental measurements of temperature-dependent DW depinning have demonstrated distributions of both depinning fields and depinning times, revealing thermally activated behavior and deviations from a simple single-barrier Néel–Brown description.^113^ The combined influence of thermal noise and material disorder may ultimately determine the practical operating window of racetrack technologies. Yet these stochastic events are often treated primarily through numerical simulations.^114^ Experimental studies capable of quantifying thermally activated behavior under realistic operating conditions remain comparatively limited.

In this context, the reliability methodologies developed for SOT-MRAM provide a useful starting point. Because SOT-MRAM and DWM share a three-terminal architecture and rely on current-induced magnetic dynamics, established metrics such as write-error rate (WER), BER, endurance, and retention can provide useful benchmarks for DWM reliability.^57,71,115^ Recent SOT-MRAM demonstrations have achieved write-error rates as low as 10^–6^, illustrating the degree of statistical reliability required for practical magnetic memories.^71,115,116^ At the same time, recent SOT-MRAM work has demonstrated more than 10^12^ write cycles while maintaining tunable retention extending beyond 10 years, highlighting the importance of jointly evaluating endurance and thermal stability (also see Table 1).^115,116^ However, these standard metrics are insufficient to fully describe DWM reliability. As the operational principles of DWMs are based on the continuous motion of domain walls rather than conventional switching mechanisms, novel methodologies for accurately assessing the effective BER need to be developed.

### 4.3 Domain-Wall Positioning Precision

Information storage in racetrack memory depends on the ability to position domain-walls accurately. Every read and write operation ultimately relies on the assumption that magnetic domains can be transported to predetermined locations with sufficient precision. In practice, achieving this objective is challenging. Domain-walls are extended magnetic objects rather than rigid particles. Their internal structure evolves during motion, and their interaction with defects can produce unpredictable displacement errors. This raises an important question: what level of positional precision is fundamentally achievable in realistic racetrack systems? Despite its obvious technological importance, this question remains largely unanswered.

To date, geometrical notches and other structural modifications have been widely proposed as effective approaches for achieving precise control of domain-wall motion.^26,77,101^ However, these engineered lateral potential landscapes may also become a source of information loss when multiple domain-walls are present within the racetrack. For example, when two nearby domain-walls propagate simultaneously, the leading wall will encounter a local potential barrier before the trailing wall. This difference in interaction timing can gradually reduce the spacing between adjacent domain walls, potentially causing domain walls to merge, which could result in information loss or bit-positioning errors.^76,103^ Such effects become increasingly critical as device dimensions and bit-spacing are scaled down. To further advance domain-wall based spintronic devices, it will be crucial to not only study the average displacement of domain-walls but also on the statistical distribution of their final positions under realistic operating conditions. Understanding these stochastic variations may ultimately determine the practical limits of racetrack density, positioning accuracy, and operating speed.

Despite these challenges, advances in electrical readout integration at the nanoscale may represent the most critical step toward practical racetrack devices. Next investigations should therefore place greater emphasis on electrical readout architectures, sensing margins, stabilities, and fabrication strategies at the nanoscale racetrack devices, rather than focusing exclusively on domain-wall motion.

### 4.4 Beyond Conventional Domain-Wall-Based Memory and Logic

The continuous and dynamical nature of domain-wall motion in racetrack devices also offers a unique opportunity to move beyond traditional binary memory and logic toward neuromorphic computing. Unlike conventional magnetic memories, in which information is primarily encoded in discrete and stationary magnetic states, domain-wall-based spintronic devices provide additional degrees of freedom through the position, spacing, and dynamics of domain-walls. These physical variables can represent analog synaptic weights, temporal states, and neuronal activity, enabling computation directly in the magnetic domain.^97,117,118^

A particularly attractive approach is to exploit electrical readout from domain-wall devices to convert their magnetic configuration into an analog signal. Experimentally, nonvolatile analog synaptic weights have been demonstrated by encoding information in the relative populations of oppositely oriented domains and controlling them through SOT-driven domain-wall motion.^118^ Similarly, SOT-driven domain-wall devices have demonstrated multiple resistive states together with experimentally realized spike and step neuron functionalities.^119^ Domain-wall magnetic tunnel junctions (DW-MTJs) have further enabled programmable multistate synapses and nonlinear neuron activation, highlighting the potential for highly integrated all-spin neuromorphic hardware.^110^ Current-induced domain-wall motion may also provide a natural platform for spiking neural networks. SOT-driven domain-wall dynamics in synthetic antiferromagnets have been used to realize leaky-integrate-fire neurons with self-reset and winner-takes-all functionality.^120^ More recently, a domain-wall racetrack integrated with MTJs experimentally demonstrated the complete integrate-fire-reset cycle over more than 100 continuous operations.^82^

Beyond analog state encoding, the domain-wall energy landscape itself can be engineered to perform neuronal functions. Durner et al. demonstrated a reconfigurable magnetic inhibitor positioned adjacent to a racetrack, whose localized stray field can selectively inhibit or promote current-induced domain-wall propagation depending on its magnetic state. By exploiting this controllable pinning and depinning behavior, the authors experimentally realized domain-wall logic and a leaky-integrate-and-fire neuronal functionality.^101^

These demonstrations suggest that the racetrack itself can serve not only as a storage medium but also as a dynamical computational platform. Controlled domain-wall propagation, pinning, depinning, and even self-driven motion could provide physical mechanisms for analog and temporal information processing, enabling the co-integration of memory, analog processing, and neuromorphic functionality within a single device architecture. From a broader perspective, such a shift from using domain walls solely as information carriers toward exploiting their intrinsic dynamics for computation could transform racetracks from conventional data-storage elements into multifunctional spintronic computing platforms, representing an important pathway beyond conventional racetrack memory.

## 5 3D Racetracks: Brief Review and Future Research Directions

Thus, far, we have mainly discussed racetrack devices with planar geometries. However, given the characteristics of domain-wall-based spintronic devices, three-dimensional (3D) structures are expected to offer significant advantages over their planar counterparts. In principle, 3D racetracks can store information along extended magnetic nano-conduits stacked vertically, enabling storage densities that are difficult to achieve using conventional planar architectures, as the original concept promised in Figure 4a.^6^ With the development of 3D lithography technologies and characterization techniques, this vision remains highly attractive today.

**Figure 4:**
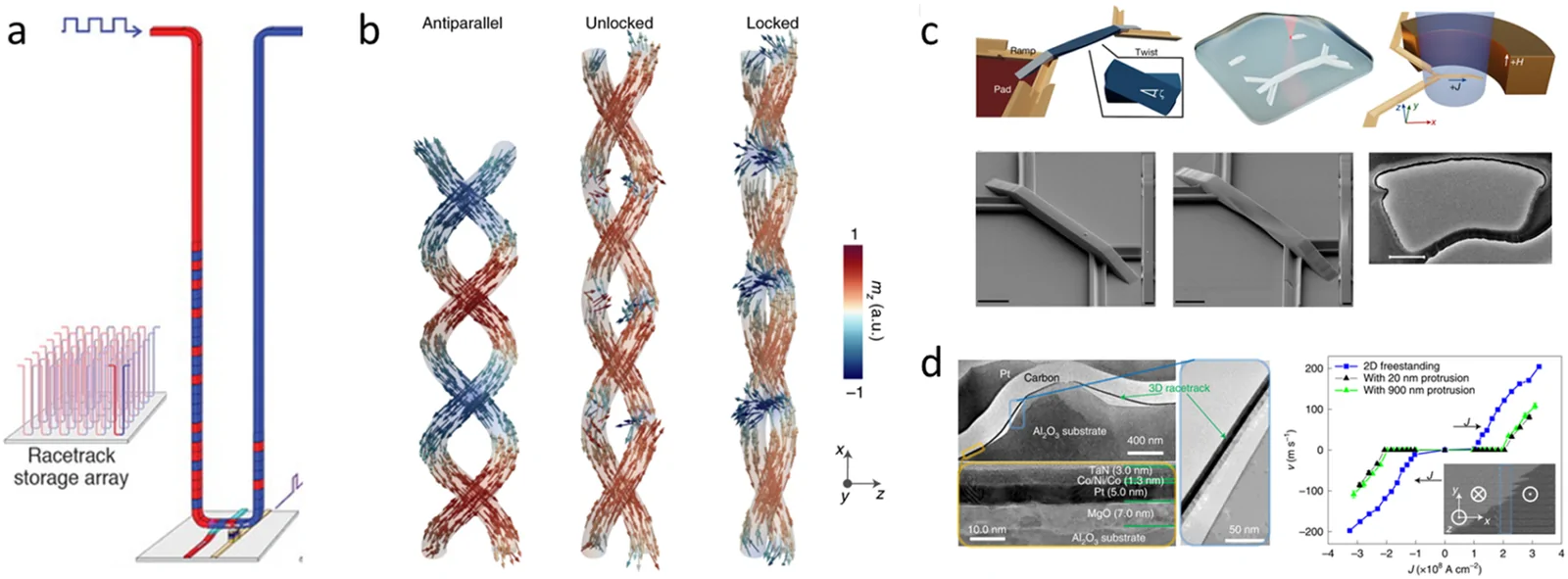
Three-dimensional (3D) racetrack devices. (a) Originally proposed concept of 3D racetrack memory device. Reproduced with permission from ref 6 Copyright [2008] Science. (b) Direct observation of spin dynamics in 3D double helix magnetic nanowires with soft-X-ray magnetic laminography. In this work, a regular array of coupled and locked domain-wall pairs in neighboring helices is identified. Reproduced from^121^. Available under a CC-BY 4.0 license. Copyright [2022] Springer Nature. (c) Fabrication of a 3D racetrack device with a torsional structure using multiphoton lithography and magnetron sputtering. With such a twisted structure, in which chiral Block-type walls are favored, nonreciprocal domain-wall motion is observed. Reproduced from^128^. Available under a CC-BY 4.0 license. Copyright [2025] Springer Nature. (d) Demonstration of 3D racetrack devices using a water-soluble sacrificial layer (Sr_3_Al_2_O_6_). With this method, free-standing racetrack devices were fabricated using a pre-patterned substrate with nearly 1 μm deep protrusions. Reproduced from^126^. Available under a CC-BY 4.0 license. Copyright [2025] Springer Nature.

Alongside fabrication advances, characterization technologies have become essential for understanding increasingly complex 3D magnetic structures. Figure 4b contains an example of showing magnetic characteristics of a 3D double helix magnetic nanowire.^80,121,122^ A foundational development was the introduction of element-specific resonant X-ray ptychographic tomography, which enabled quantitative three-dimensional imaging of nanoscale magnetic architectures with approximately 25 nm spatial resolution.^80^ Combining phase-sensitive X-ray tomography with element-specific contrast allow to reconstruct both structural and compositional information within complex 3D nanostructures. Another work investigated how 3D magnetic nanostructures generate complex magnetic stray-field distributions in the free space surrounding them.^122^ Once magnetic structures are extended into three dimensions with complex geometry, they no longer produce the relatively simple dipolar fields familiar from planar nanomagnets. Instead, geometry itself becomes a powerful tool for shaping magnetic-field textures in all three spatial dimensions.^123–125^

A major breakthrough in 3D racetrack fabrication was demonstrated through the transfer of freestanding magnetic heterostructures onto pre-patterned 3D substrates by Gu et al.^126,127^ Using a water-soluble sacrificial layer (Sr_3_Al_2_O_6_), magnetic multilayers, prepared by magnetron sputtering, were released and transferred onto substrates containing protrusions up to ∼1 μm height. This work established the feasibility of fabricating functional 3D racetrack devices and achieved a device performance of 3D systems that is as good as for conventional planar systems. Moreover, the interaction between domain-walls in a ferromagnet (Co–Ni–Co) and substrate protrusions produced a geometry-dependent propagation behavior. It enabled the selective transmission of domain walls with different chiralities, thereby realizing a domain-wall ″diode″ effect.^126^

More recently, advances in high-resolution multiphoton lithography have enabled the fabrication of truly 3D magnetic structures with complex geometries, such as twisted magnetic ribbons, where the geometrical torsion introduces an additional source of torque acting on a propagating domain-wall.^128^ Experiments showed that the interplay between geometric twisting and magnetic chirality generates effective torsional fields that strongly influence domain-wall dynamics in micron-sized 3D racetrack devices. Depending on the handedness of both the twist and the domain-wall, propagation can be either enhanced or suppressed exhibiting a diode-like behavior. Interestingly, such diode-like effect was absent in SAF racetracks. In a SAF structure where two magnetic layers are antiferromagnetically coupled, each layer contains a domain-wall with an opposite magnetic configuration. Therefore, the net torsional contribution becomes approximately zero. Such chirality-dependent filtering demonstrates how geometry itself can become an active design parameter in spintronic devices, opening possibilities for programmable logic, information routing, and advanced 3D spintronic architectures.

Despite these appealing prospects, the realization of practical nanoscopic 3D racetrack devices remains distant. Much of the existing literature focuses on demonstrating isolated aspects of 3D operation, including complex vertical domain-wall motion or curved magnetic conduits.^123–125^ While these achievements represent important milestones, they should not be confused with complete 3D device architectures.

A practical 3D racetrack system must simultaneously overcome several key challenges. Figure 5 summarizes major milestones in practical 3D racetrack devices. While conventional considerations in planar racetrack devices, such as achieving efficient domain-wall motion, precise domain-wall positioning, and stable domain-wall control, also apply to 3D racetrack devices, additional geometry-induced challenges must be addressed to realize practical 3D racetrack devices. First, domain-walls must be shifted reliably through complex geometries. Unlike planar racetracks, 3D structures introduce additional geometry-induced energy landscapes. These features may create unintended pinning sites or alter domain-wall dynamics in other undesirable ways. Second, electrical write and read operations, which are already not trivial even in planar devices, become increasingly complex in 3D geometries. Accessing individual bit locations within densely packed vertical structures requires sophisticated addressing schemes that do not compromise density advantages. The associated interconnect networks may introduce substantial overhead in both area and energy consumption. To mitigate these geometry-induced energy and area overheads, compact device designs and the development of materials with lower operating energy requirements will be essential. Third, reliable and scalable fabrication of 3D racetrack devices remains a major obstacle. Although remarkable advances have been achieved in nanofabrication, producing three-dimensionally interconnected magnetic conduits with electrical read ports remains extremely challenging. Furthermore, variations in layer thickness, interface quality, sidewall or surface roughness, and magnetic properties may become significant in complex 3D geometries. Methods for achieving uniform film quality over 3D structures are urgently needed. Moreover, the high-aspect-ratio geometry of 3D racetrack devices introduces additional challenges for thermal management. Because a minimum current density is required to drive domain-wall motion along extended nanowires, Joule heating is unavoidable during device operation. As the device density per unit chip area increases, effective heat dissipation becomes increasingly important for managing the resulting thermal load, preventing thermal degradation, maintaining reliable domain-wall dynamics, and ensuring long-term device stability. Therefore, further development of effective heat-dissipation strategies will be necessary to manage these thermal loads while maintaining reliable operation in densely packed 3D structures.

**Figure 5:**
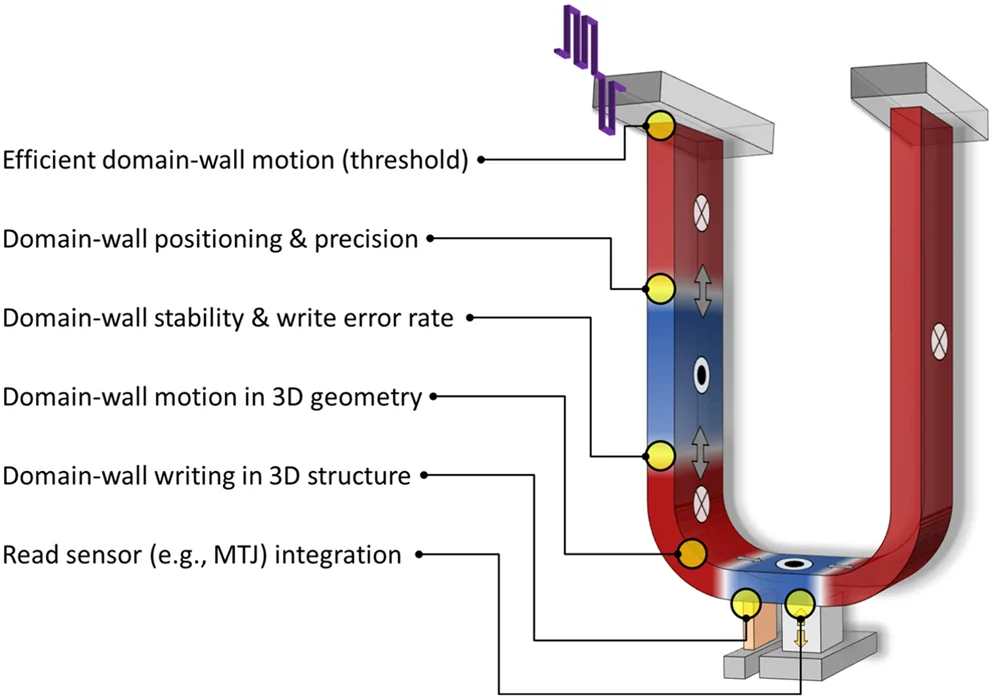
Summary of the milestones for scalable 3D domain-wall-based spintronic devices. In 3D structures, geometry-induced effects such as stray fields, domain-wall coupling, pinning, and induced magnetic properties, as well as the integration of electrical read and write ports, must be considered alongside conventional factors, including domain-wall positioning, threshold current density, and stability. Furthermore, the geometric complexity of these structures may introduce additional energy and area overheads, which must be considered in the design of devices and materials to minimize the energy required for operation.

These considerations suggest that the 3D racetrack memory may require a broader or completely novel perspective than simply extending planar architectures into the vertical dimension. Research should consider 3D racetrack concepts from a systems perspective. Demonstrations of individual physical mechanisms remain valuable, but equal emphasis should be devoted to questions of manufacturability, architecture, thermal management, and system-level performance.

## 6 Outlook

The evolution of racetrack memory over the past two decades is a remarkable success story in fundamental spintronics. Many challenges that once appeared formidable have been progressively addressed through advances in materials science, atomic-scale multilayer stack engineering, device design, and domain-wall control mechanisms. As a result, racetrack technology has reached a high level of maturity, bringing the field closer than ever to practical implementation and technological viability. In our view, the next decade should be guided by the following research priorities (Table 3).

**Table 3:** Research Priorities for Next-Generation Domain-Wall-Based Spintronics

research priorities	opportunities
standard benchmark	setting standard benchmark for the evaluation of racetrack device performance:
	velocity, threshold current density, position accuracy, bit-error rate, cycle-to-cycle variability, and endurance.
detection of domain-wall dynamics in nanoscale	understanding domain-wall dynamics in nanoscale:
	development of novel detection techniques with superior spatiotemporal resolution, direct integration of electrical detection sensors (e.g., MTJ, Hall bar).
system-level integration	system-level integration and performance comparison:
	optimization of device architectures; integrating MTJs, peripheral circuits, control units, and performance comparison with other current state-of-art memories.
novel materials and device schemes	exploring novel materials and device schemes:
	understanding domain-wall dynamics and physics in novel materials, novel device schemes for efficient control of domain-wall motion in nanoscale.
beyond racetrack memory	beyond racetrack memory:
	research on emerging devices beyond memory – artificial intelligence, cryptography, in-memory computing, edge computing, neuromorphic computing.

### 6.1 Research Priority 1: Standard Benchmarks

For many years, achieving higher domain-wall velocities has been a central objective. However, the racetrack community lacks standardized metrics. Critical performance indicators, such as cycle-to-cycle variability, positional accuracy, retention, endurance, and BER are often not clearly addressed in nanoscopic racetrack devices.

The establishment of common benchmarking protocols would greatly facilitate meaningful comparisons between competing materials systems and device architectures. Future studies should focus on statistical distributions and reliability metrics rather than relying solely on representative device demonstrations – such as velocity and threshold current density.

### 6.2 Research Priority 2: Detection of Nanoscale Domain-Wall Dynamics

An important message in this perspective is the limited understanding of domain-wall behavior at technologically relevant dimensions. Addressing this challenge will require new experimental methodologies with higher spatial and temporal resolution. Electrical measurement techniques capable of tracking domain-wall motion with nanometer-scale sensitivity and high temporal resolution would provide valuable information. Such methods could enable large-scale statistical studies that are difficult to achieve in the nanoscale using existing imaging approaches. The development of advanced characterization techniques and tools should therefore be viewed as a central research objective rather than a supporting activity. In particular, this will be a critical turning point for 3D racetracks.

### 6.3 Research Priority 3: System-Level Integration

Given the increasing maturity of racetrack technology, at least in planar structures, the field should dedicate more attention to MTJ integration, sensing architectures, fabrication compatibility, and circuit-level considerations. Successful solutions in these areas would immediately elevate the technological relevance of racetrack devices and accelerate their transition toward practical applications. Furthermore, comparisons of performance with other memory technologies across multiple aspects will be required.

### 6.4 Research Priority 4: Novel Materials and Device Schemes

Historically, advances in material development and device design have been the primary drivers of major breakthroughs in racetrack technologies. As the discovery and synthesis of novel magnetic materials, such as van der Waals and topological quantum materials, continue to accelerate, progress will significantly depend on understanding and exploiting domain-wall behavior within these emerging material systems. Material properties alone do not guarantee superior device performance; innovative device architectures are often required to fully utilize their potential and promise reliable operation. Technologically attractive device architectures may impose constraints that influence material selection. Consequently, optimizing materials and devices independently may not yield the best overall system performance. Co-optimization strategies that simultaneously consider materials, device architectures, circuits, and manufacturing processes are therefore likely to play an important role in the development of racetrack technologies.

### 6.5 Research Priority 5: Beyond Racetrack Memory

The original goal of racetrack research has often been framed as replacing existing memory technologies. While this high-density and high-speed memory concept remains appealing, it may not represent the most realistic pathway toward technological impact. Emerging computing paradigms demand specialized hardware capable of supporting artificial intelligence, edge computing, in-memory processing, inference, cryptography, and neuromorphic functionality. Racetrack devices possess unique characteristics, including nonvolatility, shift-register type operation, stochasticity, high-speed, spatial information processing, and dynamical magnetic states, that may be particularly valuable in these applications. The success of racetrack technologies may therefore depend not only on competing with established memories but also on enabling functions beyond conventional computational elements.

## 7 Conclusion

Nearly two decades after its introduction, racetrack memory remains one of the most exciting concepts in spintronics. The field has significantly advanced our understanding of domain-wall driving mechanisms, domain-wall dynamics, interface effects, geometrical/dimensional effects, and magnetic materials. These achievements have elevated racetrack memory from a conceptual idea into a scientifically mature research area. Research should evolve to focus on questions of reliability, precision, readout, integration, manufacturability, system architecture, and benchmarking. In particular, nanoscale domain-wall dynamics remain largely unexplored, despite their central importance for technological implementation. The development of integrated readout schemes, standardized quantitative reliability metrics, advanced nanoscale characterization techniques, novel computational elements, and scalable 3D architectures will likely define the next phase of domain-wall-based spintronic devices. Success in these areas could finally bridge the gap between fundamental spintronic phenomena and practical information technologies. Altogether, the future of racetrack memory and related devices appears highly promising.
